# Does Pathological Stage and Nodal Involvement Influence Long Term Oncological Outcomes after CROSS Regimen for Adenocarcinoma of the Esophagogastric Junction? A Multicenter Retrospective Analysis

**DOI:** 10.3390/cancers13040666

**Published:** 2021-02-07

**Authors:** Stefano de Pascale, Paolo Parise, Michele Valmasoni, Jacopo Weindelmayer, Fabrizia Terraneo, Chiara Alessandra Cella, Simone Giacopuzzi, Andrea Cossu, Simonetta Massaron, Ugo Elmore, Stefano Merigliano, Uberto Fumagalli Romario, on behalf of the Italian Society for the Study of Esophageal Diseases (SISME)

**Affiliations:** 1 Department of Surgery, European Institute of Oncology IRCCS, 20141 Milan, Italy; uberto.fumagalliromario@ieo.it; 2 Department of Gastrointestinal Surgery, San Raffaele Scientific Institute, 20132 Milan, Italy; parise.paolo@hsr.it (P.P.); cossu.andrea@hsr.it (A.C.); massaron.simonetta@hsr.it (S.M.); elmore.ugo@hsr.it (U.E.); 3 Department of Surgical, Oncological and Gastrointestinal Sciences, University of Padova, Clinica Chirurgica 3, 35128 Padova, Italy; michele.valmasoni@unipd.it (M.V.); stefano.merigliano@unipd.it (S.M.); 4 General and Upper GI Surgery Division, University of Verona, 37129 Verona, Italy; jacopo.weindelmayer@aovr.veneto.it (J.W.); simone.giacopuzzi@univr.it (S.G.); 5 Department of Radiotherapy, Spedali Civili di Brescia, 25123 Brescia, Italy; fabriziaterraneo@virgilio.it; 6 Gastrointestinal Medical Oncology and Neuroendocrine Tumors, European Institute of Oncology (IEO), 20143 Milan, Italy; chiaraalessandra.cella@ieo.it; 7 Departement of Molecular and Translational Medicine, University of Brescia, 25121 Brescia, Italy

**Keywords:** gastroesophageal cancer, CROSS regimen, esophagogastric junction cancers, neoadjuvant therapy

## Abstract

**Simple Summary:**

Chemoradiotherapy according to CROSS regimen is the standard of care for locally advanced esophageal cancer. The studies conducted on this topic have demonstrated the benefits of this type of treatment particularly for squamocellular cancers. Its application for adenocarcinoma has evidenced different results and few studies have investigated its role for adenocarcinomas of esophagogastric junction. Our intent is to evaluate the relation between pathological (yp) stage after CROSS regimen followed by surgery for adenocarcinoma of cardia and overall (OS) and disease-free survival (DFS) in a retrospectively analyzed group of patients. Sites of relapse after surgery were also analyzed. Our results evidenced no differences in term of OS and DFS according to different pathological response after chemoradiotherapy and surgery. Further analyses could be performed to identify the histological and molecular characteristics of these tumors and predict the efficacy of systemic therapy identifying patients who can most benefit from this type of treatment.

**Abstract:**

**Background:**After the results reported by the “Chemoradiotherapy for esophageal Cancer Followed by Surgery Study” (CROSS) trial, neo-adjuvant chemoradiotherapy became the standard treatment for locally advanced cancers of esophagus and gastroesophageal junction (GEJ). Excellent results were reported for squamocellular carcinomas (SCCs). Since the advent of the CROSS regimen, the results of surgery for esophageal adenocarcinomas (EAC) have cast some doubts about its efficacy on overall survival (OS) even in the presence of local response. This study evaluated the relation between pathological (yp) stage after CROSS regimen followed by surgery for adenocarcinoma of cardia and overall (OS) and disease-free survival (DFS). Sites of relapse after surgery were also analyzed. **Methods:** Patients submitted to the CROSS regimen for locally advanced EAC of the cardia followed by transthoracic esophagectomy were analyzed. Actuarial OS and DFS were analyzed and stratified according to yp stage. The site of relapse, distal and local, was also analyzed. **Results:** The study included 132 patients. The 50-month OS and DFS were 45% and 6.7%, respectively. No differences emerged analyzing OS according to yp stage. Time to relapse was significantly longer for yp Stage I and II, and for yp N0, compared with yp N+. Recurrence occurred in 48 cases (36.3%) with a 9 months median time to relapse. Local and distal relapse were 10 (7.5%) and 38 (28.7%) cases, respectively (*p* ≦ 0.001). **Conclusions:** Pathological stage after CROSS regimen does not relate to OS and DFS. Time to recurrence is significantly longer for yp Stages I and II and ypN0. Chemoradiotherapy in a neoadjuvant setting may influence the site of relapse, significantly reducing local recurrences.

## 1. Introduction

With over 450,000 new diagnoses per year, esophageal cancer (EC) is the 8th most common neoplasm globally, and incidence has increased in recent decades. EC has a poor prognosis, making it the 6th most lethal malignancy, with an estimated 400,000 deaths per year [1]. Surgical resection is the standard treatment for cancers of gastroesophageal junction (GEJ). The results of primary surgery appear to be unsatisfactory in locally advanced diseases due to early local recurrence and metastatic spread, which often lead to a high rate of non-curative resections due to microscopic residual tumors (R1). According to these data, interest has focused on the benefits of neoadjuvant chemotherapy (NAC) with or without radiation therapy (RT) [2] before surgery. The MAGIC trial demonstrated that it may be possible to improve overall survival (OS) with perioperative chemotherapy without increasing surgical morbidity; the multimodal approach has subsequently become the routine treatment for GEJ cancers [3]. Different approaches were evaluated thereafter, testing the safety, feasibility and efficacy of different neoadjuvant or perioperative regimens, including perioperative chemotherapy (CT) and neoadjuvant chemoradiotherapy (NACRT), which were compared to surgery alone. In 2011, a wide meta-analysis comparing multimodal treatments for esophageal cancer suggested significant improvement using neoadjuvant therapies, with NACRT having the lowest association with all-cause mortality (hazard ratio (HR): 0.78; 95% confidence interval (CI): 0.70–0.88) compared with chemotherapy (HR: 0.87; 95% CI: 0.79–0.96) [4]. Concurrent NACRT has become the standard treatment for locally advanced GEJ cancers [5,6]. These results were further strengthened by the results reported by the CROSS trial in 2012 [7], and this regimen has become the standard of care for patients affected by locally advanced EC [8].

The pathological response and pathological staging after treatment are the principal elements that influence clinicians to suggest the use of NACRT. This type of approach for locally advanced GEJ cancers has the intent of downstaging locally advanced tumors, obtaining an OS and a disease-free survival (DFS) similar to those of naïve patients affected by more localized esophageal neoplasms. Although this therapeutic combination has yielded excellent results for squamocellular carcinomas (SCCs), less favorable results for EAC were reported, casting some doubts about its efficacy on OS even in the presence of a local response. 

To evaluate the results of this regimen in an unselected population of patients, we planned this retrospective study. Its primary aim was to analyze the relation between pathological stage after the application of the CROSS regimen for locally advanced adenocarcinomas of the cardia, OS and DFS. The secondary aim is to analyze the sites of relapse after surgery to explain the reasons for failure.

## 2. Results

The general characteristics and surgical approaches of the 132 included patients are reported in Table 1. The clinical assessment (c) of the tumors, nodes and metastases (TNM) for all patients was cTNM ≥ T3N0 or any N+. The postoperative complications, clustered according to the Clavien Dindo classification [9], were grade 1 or 2 in 28% of patients, and grade 3A, 3B, 4A and 4B for 15%, 10%, 4.5% and 2% of patients, respectively. Thirty-days and 90-days postoperative mortality rates were 0.8% and 1.5%, respectively. 

The rate of R0 and R1 resections, median lymph nodes harvested and yp stage are reported in Table 2. The median time to follow-up was 14 months (range 4–51 months, iq range 6–26). At the final follow-up, 84 patients were relapse-free.

**Figure 1:**
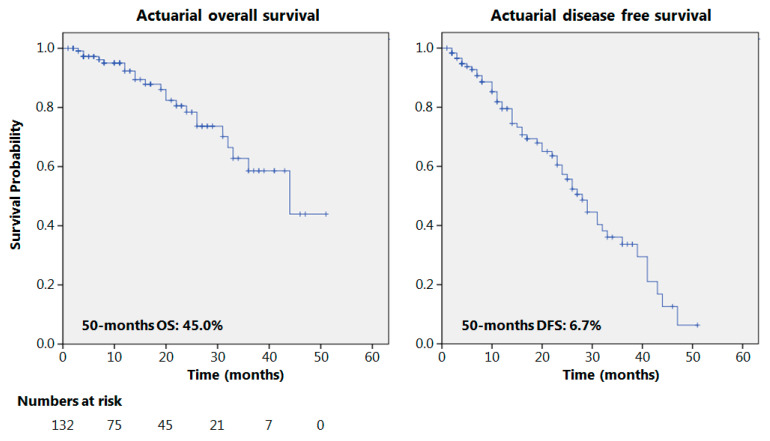
Overall and disease-free survival.

The actuarial 50-month OS and DFS were 45% and 6.7%, respectively, as reported in Figure 1. Seventy patients presented a 12-month follow-up or longer with recurrence in 36 cases (51.4%); 38 patients had a 24-month follow-up or more; and relapses were observed in 20 cases (52%). The results of some of the principal oncological studies on NACRT for esophageal adenocarcinomas, in the current literature, are reported in Table 3 and compared to the results obtained in the present study.

Local and distant recurrence occurred in 7.5% and 28.7% of patients, respectively (*p* < 0.001). The principal sites of distal relapse were liver (23.6%), lung (21%), bone (15.7%) and brain, pleura, peritoneum, and distal nodes (5.2%). The median time to recurrence was 9 months (range 4–39 months). Recurrences are also clustered according to yp stage in Table 4.

**Figure 2:**
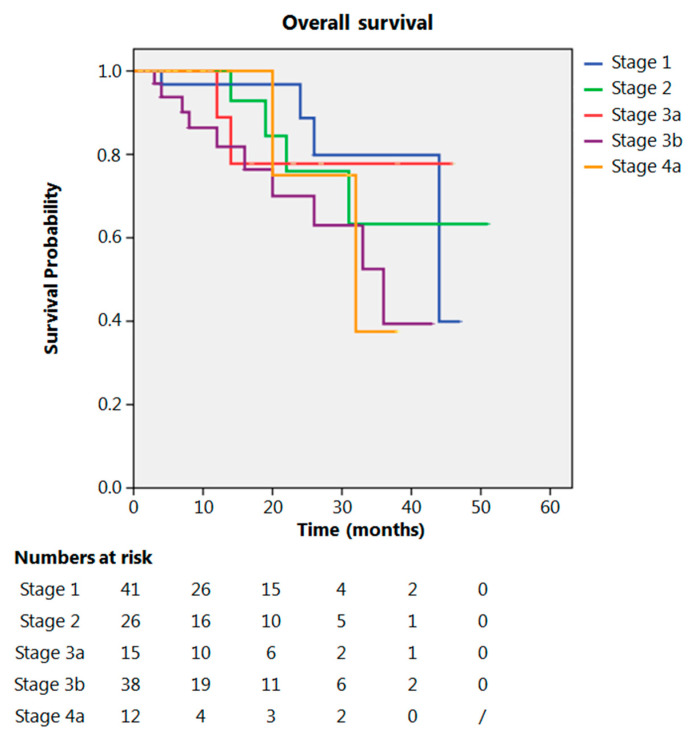
Overall survival according to yp stage.

**Figure 3:**
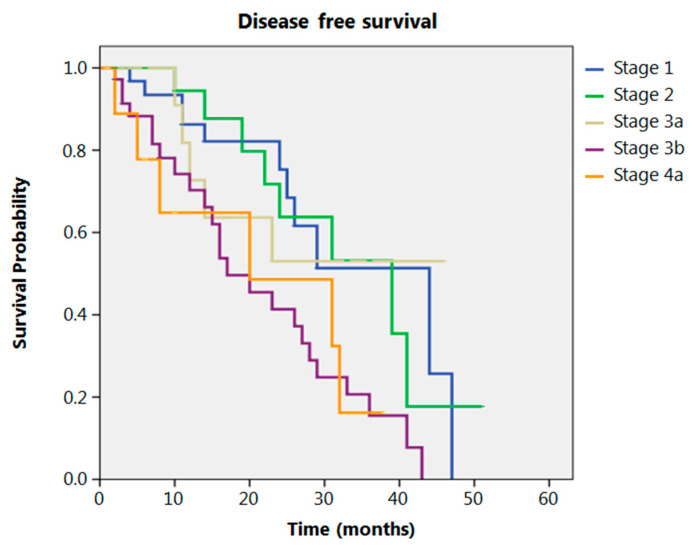
Disease free survival according to yp stage.

**Figure 4:**
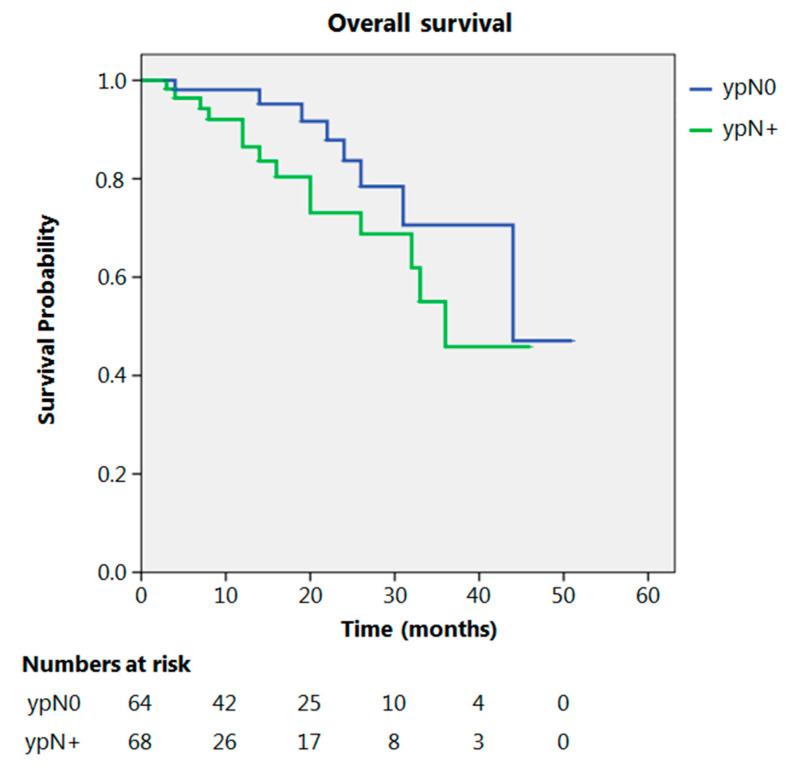
Overall survival according to ypN.

**Figure 5:**
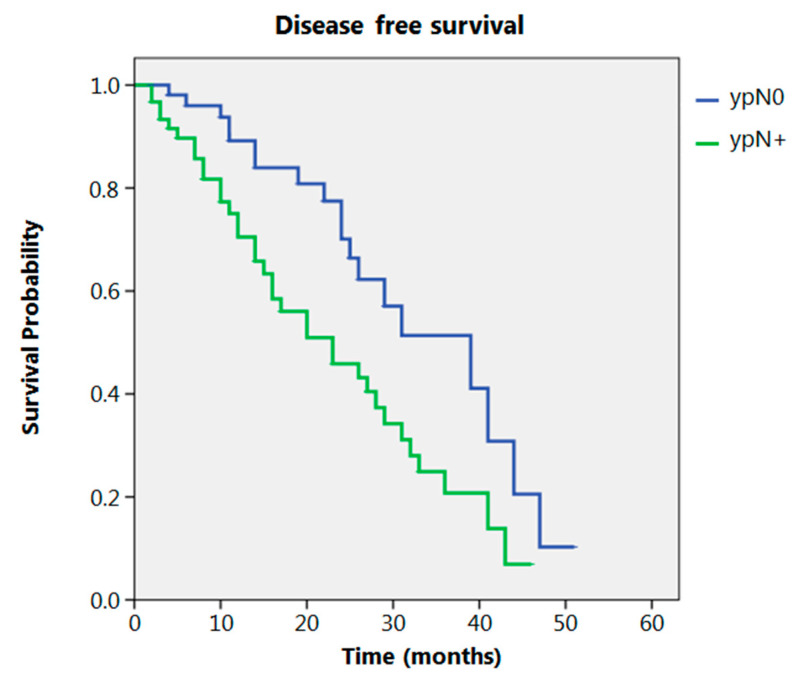
Disease free survival according to ypN.

Even after clustering the pathological stages after the CROSS regimen, no differences emerged in terms of OS (*p* = 0.315) (Figure 2). Patients with yp Stage I and II had a significantly longer time to recurrence in comparison to all other stages (*p* = 0.013) (Figure 3). No differences in terms of OS emerged from the comparison of ypN0 patients with ypN+ patients, (*p* = 0.117) (Figure 4). The 50-month DFS for these two groups of patients was 11% and 8%, respectively, for ypN0 and ypN+, and time to relapse was significantly longer for the ypN0 group (*p* = 0.005) (Figure 5).

## 3. Discussion

Several previous studies have addressed the importance of integrated therapies for the treatment of locally advanced esophageal cancers. In 1996, Walsh [16] demonstrated that the median OS of patients affected by adenocarcinoma of the esophagogastric junction (AEGJ) appeared to be significantly higher (16 versus 11 months) when they were treated with multimodal therapy rather than by surgery alone. Several years after, the CROSS trial demonstrated an improved result for NACRT before surgery rather than surgery alone [7]. In 2015, Shapiro [10] reported the long-term results of the CROSS trial that confirmed a high rate of pathological responses either for SCC and EAC, and good results for OS and DFS after combined treatment. This is now a routine treatment for patients affected by locally advanced esophageal neoplasms. However, the separated analysis of data for patients with SCC and EAC indicates a significantly better result for the first group. The application of this regimen to the EAC group has a still favorable, but reduced, impact on OS, compared with patients treated with surgery alone. The OS reported for EAC after NACRT in the CROSS trial was 46% at 60 months; the OS results for our unselected population of patients treated for locally advanced adenocarcinoma of the EGJ with this regimen are similar. There is a significant difference, however, between data from our study and data obtained from the Dutch study regarding DFS: our results report 6.7% of patients without relapse after 50 months with most recurrences being distant compared to a 60-month DFS of 42% in the CROSS study. Other studies on the results of NACRT for EC have been conducted during the last few years (Table 3). Koch compared preoperative chemotherapy with the CROSS regimen for patients with adenocarcinoma of the esophagus and EGJ: 60-month DFS for patients treated with NACRT was 8% [15], similar to that obtained in our experience. Better results in terms of DFS were obtained in other studies [10,11,12,13,14]. 

This significant difference in terms of DFS between the two studies (ours and Koch’s) and the other studies is difficult to explain. One reason could be the small number of patients affected by adenocarcinoma of the EGJ in the CROSS trial (22%), in Anderegg’s (26.7%) and Visser’s analysis (20%). Patients with adenocarcinoma of the EGJ have different lymphatic spread and Lauren’s histotype [17]; patients with adenocarcinoma of the EGJ have a higher incidence of nodal metastases to the celiac trunk compared to patients with EAC. These metastases have been demonstrated to be a significant negative predictive factor for long-term oncological prognosis [18]. 

Differences in Lauren’s histotype might also explain the variation in survival. Lauren’s histotype and signet ring cell differentiation (SRC) have been considered as prognostic parameters in several analyses. A retrospective study published in 2019, conducted on patients affected by carcinoma of the EGJ, indicated that there is no statistically significant difference in OS according to SRC status following NACT or NACRT [19]. However, the DFS of patients with an SRC component is significantly better after NACRT compared to NACT, demonstrating the importance of a local therapy effect in this subset of patients with negative prognosis. Similar data are also present in our study where patients treated with NACRT independently of the presence of SRC have significantly better local control, but without a significant advantage in terms of OS. 

Our data demonstrate that there is no difference in the 50-month OS according to yp stage (8th TNM classification). yp stage, therefore, does not seem to stratify patients in terms of long-term oncological outcomes. Similar results were obtained by other groups. In 2018 Sisic [20] analyzed the survival of patients submitted to esophagectomy after preoperative treatment; in his experience ypT did not stratify patients for OS into ypT1 and ypT2 groups. The only significant prognostic difference was noted for the ypT4 group, which had a significantly worse prognosis compared to other groups of patients. The exclusion of cT4 patients in several studies reported in Table 3 might be another reason to explain the difference in DFS. In our analysis, 11% of patients had a cT4 neoplasm and this could negatively influence the long-term oncological outcomes, particularly for DFS. 

Regarding the N parameter in our study, no significant difference was obtained when comparing ypN0 patients with ypN+ patients in terms of OS. Others found a better prognosis for ypN0 compared to ypN+ patients [16]. Our study shows that nodal metastases after NACRT are a predictor of a significantly earlier relapse rate. 

Depypere recently evaluated the impact of lymph node response (LNR) after NACRT as a prognostic factor for OS and DFS. Patients with LNR related to NACRT have OS and DFS similar to pN+ patients [21]. 

The rate of distant metastases, in our experience, is significantly higher than the rate of local relapse, demonstrating an improved local control for NACRT but a low efficacy for systemic disease control. Similar results have recently been reported by others for EA after NACRT. An opposite efficacy was reported for patients with SCCs who received the same treatment [22]. 

It is interesting to note that, although the numbers are small and with short actuarial analysis of DFS (median follow-up of 14 months), patients with yp Stage IIIA and IIIB nonetheless have a higher rate of distant relapse in comparison to earlier pathological stages. This evidence requires a longer follow up with the analysis of site of recurrence in a larger series of patients. 

In our study, 49% of patients were treated with a minimally invasive technique, a percentage comparable to that reported in the Esodata group of patients [23]. 

Differences in overall survival cannot be linked to different surgical approaches, either open or minimally invasive. In a recent retrospective analysis, conducted on patients treated with a minimally invasive esophagectomy after the CROSS regimen, Lubbers et al. reported favorable results in terms of overall survival and of postoperative complications. Other authors reported similar results for NACRT regimens followed by minimally invasive surgery [24]. A meta-analysis published in 2016 indicated that, from an oncological point of view, the median number of lymph nodes harvested and the rate of R0 resections were similar when a totally minimally invasive Ivor Lewis (TMIIL) esophagectomy was compared to other types of esophagectomies [25].

The main limitations of our study are its retrospective and multicenter nature with the possibility of a selection bias mainly after the FLOT regimen (docetaxel, oxaliplatin, leucovorin, and 5-fluorouracil) became on option for these patients. Since then, NACRT was mainly indicated in the case of bulky tumors. 

In addition, the OS and DFS are actuarial and 62 patients (45%) had a follow-up shorter than 12 months. The short follow-up is a weakness of this study even if the total number of patients with recurrence is significant, with most recurrences occurring early. 

## 4. Material and Methods

Patients affected by locally advanced adenocarcinoma of the cardia classified as Siewert I and II (cT ≥ 3 N0 or any N+ or T2N+, according to 8th Edition of the AJCC-TNM classification for Esophageal cancer) and treated with the CROSS regimen before undergoing Ivor Lewis esophagectomy between January 2014 and January 2019 at five Italian high-volume centers for esophageal surgery associated with the SISME (Italian Society for the Study of Esophageal Diseases) were analyzed in this study.

**Figure 6:**
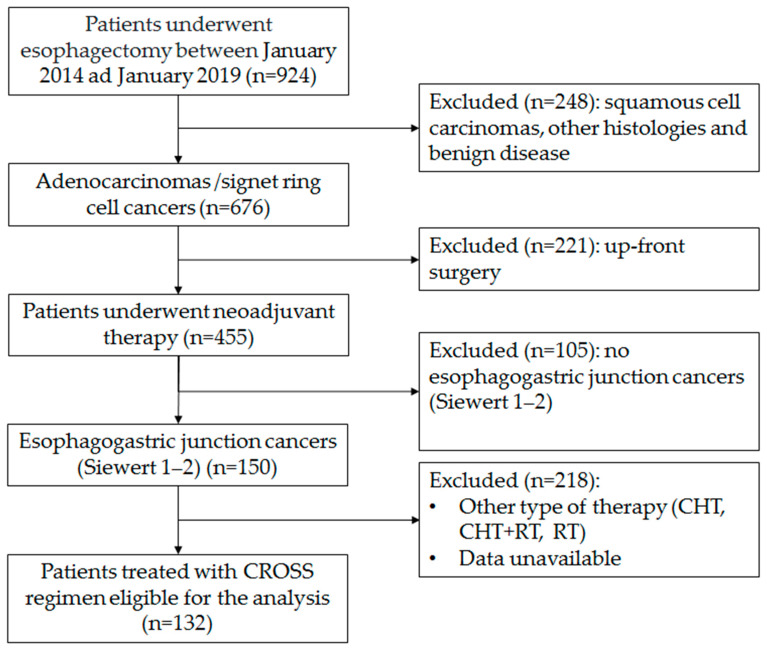
Flow-chart of the enrolment procedure.

The flowchart of the enrolment procedure is reported in Figure 6. All centers involved in this analysis have a case load for esophagectomy of over 40 cases/year. Patients submitted either to primary esophagectomy or esophagectomy for benign disease, squamous cancer, or other histology or adenocarcinoma in sites other than the esophagogastric junction were excluded. Patients submitted to the McKeown procedure or patients submitted to other type of neoadjuvant treatment were also excluded. All patients were clinically staged with endoscopy and computerized tomography (CT) scan, with a selective use of endoscopic ultrasound (EUS) and positron emission tomography (PET) scan, both in the staging and in the restaging setting. All treatment plans were discussed with the multidisciplinary esophageal tumor board. All included patients were treated with the CROSS regimen (carboplatin and paclitaxel for 5 weeks with concurrent radiotherapy of 41.4 Gy in 23 fractions). Restaging was performed 6 to 8 weeks from the end of the neoadjuvant treatment. Surgical procedures were conducted with open, hybrid or totally minimally invasive approaches according to the choice and expertise of individual surgeons in a period of 8–12 weeks after completion of radiotherapy. Since 2018, in most centers, the FLOT regimen has been progressively introduced as an alternative standard therapy for this neoplasm. Even if CROSS has remained a reference therapy, in recent years it has been considered mainly for patients in which a significant tumor shrinkage is aimed at for an R0 resection.

All of the specimens were staged by the dedicated pathologists of the different centers involved in the multicenter study.

Data were retrospectively analyzed from prospectively collected databases. The American Society of Anesthesiologists (ASA) score and the Charlson Comorbidity Index (CCI) score were evaluated. Postoperative complications were clustered according to Clavien Dindo classification. The actuarial OS and DFS were analyzed for the whole population and patients were categorized according to yp stage for adenocarcinoma as described in the 8th edition of TNM. The sites of relapse were defined as local (anastomotic and locoregional lymph nodes) or distant (lung, peritoneal, distal lymph nodes, bone, liver, brain, pleura and other sites). The time to relapse was also analyzed. 

DFS was calculated from the date of surgery to the date of first recurrence, and OS was calculated as the date of cancer-related death. The Kaplan–Meier method was used to estimate actuarial OS and DFS with the log-rank test to ascertain significance (*p* < 0.05). Median and range were used where necessary. A chi-squared test was also used. Statistical analysis was performed using IBM SPSS Statistics for Windows, Version 21.0. Released 2012. Armonk, NY: IBM Corp. software. 

## 5. Conclusions

This analysis reports the results obtained by applying the CROSS regimen during a 5-year period in five Italian high-volume centers for esophageal surgery. Our data are similar to those reported in the CROSS trial in terms of OS. DFS is lower in comparison to the results obtained by other authors. This different result should be considered for further specific sub-analysis related to clinical stratification of patients who are candidates for the CROSS regimen. The clustering of patients according to yp stage (8th TNM classification) does not evidence differences in terms of OS and DSF. Time to recurrence is influenced by the use of NACRT, particularly for yp Stage I and yp Stage II and ypN0 patients. Local control is effective; however, the CROSS regimen does not appear to significantly change the natural history of this disease in terms of oncological prognosis. 

Multiple multicenter studies are ongoing to compare the results of NACRT with the CROSS regimen and NAC with the FLOT regimen for these cancers. These studies may clarify whether other therapies will improve the systemic control of the disease. 

Further analyses should be performed to identify the histological and molecular characteristics of these tumors to predict the efficacy of systemic therapy and identify patients who can most benefit from this type of treatment.

## Figures and Tables

**Table 1:** General characteristics and surgical approach.

General Characteristics	132 Pts
Male/Female	115/17
Age, Median (range)	63.6 (28–82)
ASA score, Median (range)	2 (1–3)
1, n° (%)	2 (2)
2, n° (%)	73 (55)
3, n° (%)	56 (42)
4, n° (%)	1 (1)
Charlson comorbidity index, median (range)	2 (0–8)
BMI (Kg/m^2^), median (range)	28 (18–34)
Signet ring cell carcinoma, n° (%)	24 (18%)
Grading	
G1, n° (%)	11 (8)
G2, n° (%)	66 (50)
G3, n° (%)	55 (42)
Clinical T-stage	
cT1b, n° (%)	3 (2)
cT2, n° (%)	19 (14)
cT3, n° (%)	96 (73)
cT4, n° (%)	14 (11)
Clinical N-stage	
cN0, n° (%)	12 (9)
cN1, n° (%)	78 (59)
cN2-3, n° (%)	42 (32)
Siewert Classification	
1, n° (%)	78 (59)
2, n° (%)	54 (41)
Surgical Technique	132 pts
Open Ivor Lewis, n° (%)	16 (12)
Hybrid Ivor Lewis, n° (%)	51 (39)
Totally Minimally Invasive Ivor Lewis (TMIIL), n° (%)	65 (49)

**Table 2:** Oncological outcomes.

Oncological Outcomes	132 Pts
Lymph nodes harvested, median (range)	25 (8–65)
lymph nodes N0/N+, n° patients %	66/56
R0/R1, n° patients (%)	129/3 (98/2)
Tumor regression grade (Mandard)	
1, n° (%)	18 (14)
2, n° (%)	26 (20)
3, n° (%)	57 (43)
4, n° (%)	28 (21)
5, n° (%)	3 (2)
yp Stage I, n° (%)	41 (31)
yp Stage II, n° (%)	26 (20)
yp Stage IIIA, n° (%)	15 (11)
yp Stage IIIB, n° (%)	38 (29)
yp Stage IVA, n° (%)	12 (9)

**Table 3:** Oncological results of current literature.

Authors	Year	Type of Study	ADC EGJ * n°/All Neoadjuvant Treatment	N° pts CROSS/n° pts NACRT	Mandard 1	Median F-UP Time (Months)	OS (NACRT Group)	DFS (NACRT Group)
Shapiro et al. [10]	2015	Prospective randomized	39/178	178/178	23%	84	45% ^†^	44% ^†^
Anderegg et al. [11]	2017	Retrospective	47/313	176/176	15.1%	42	40% ^†^	40% ^†^
Goense et al. [12]	2017	Retrospective PS ^§^	-/168	84/84	17.9%	21	50% ^¥^	55% ^¥^
Favi et al. [13]	2017	Retrospective PS ^§^	80/80	40/80	23%	n.a	42% ^¥^	n.a
Visser et al. [14]	2018	Retrospective PS ^§^	55/262	14/131	15% ^‡^	47	33% ^†^	39% ^†^
Koch et al. [15]	2019	Retrospective	53/104	53/53	-	17	9% ^†^	8% ^†^
Present study	2020	Retrospective	132/132	132/132	14%	14	45%	6.7%

* Adenocarcinoma of the esophagogastric junction ^§^ Propensity score; ^†^ Evaluation at 5 years; ^¥^ Evaluation at 3 years ^‡^ Percentage related to the whole neoadjuvant chemoradiotherapy (NACRT) group (*n* = 131).

**Table 4:** Recurrence pattern according to yp stage.

yp Stage (n° Pts)	Local Recurrence, n° Pts (%)	Distant Recurrence, n° Pts (%)
I (41)	2 (4.8)	8 (19.5)
II (26)	1 (3.8)	5 (19.2)
IIIa (15)	2 (13.3)	4 (26.6)
IIIb (38)	2 (5.2)	20 (52.6)
Iva (12)	0	4 (33.3)

## Data Availability

Data are recorded and anonymized in a prospective database.
